# Comparative Analysis of Academic, Behavioral, and Psychophysiological Variables in Male and Female Vocational Training Students

**DOI:** 10.3390/children11070851

**Published:** 2024-07-13

**Authors:** Vicente Javier Clemente-Suárez, Ana Isabel Beltrán-Velasco, Daniel Mendoza-Castejón, Stephanie Rodríguez-Besteiro, Francisco López-Varas, Alexandra Martín-Rodríguez

**Affiliations:** 1 Faculty of Sport Sciences, Universidad Europea de Madrid, 28670 Villaviciosa de Odón, Spain; vctxente@yahoo.es (V.J.C.-S.); daniel.mendoza@universidadeuropea.es (D.M.-C.); stephanie.rodriguez@universidadeuropea.es (S.R.-B.); francisco.lopez@universidadeuropea.es (F.L.-V.); 2 Grupo de Investigación en Cultura, Educación y Sociedad, Universidad de la Costa, Barranquilla 080002, Colombia; 3 Psychology Department, Faculty of Life and Natural Sciences, Nebrija University, 28240 Madrid, Spain; abeltranv@nebrija.es; 4 Faculty of Applied Social Sciences and Communications, UNIE, 28015 Madrid, Spain

**Keywords:** gender differences, vocational training, academic achievement, behavioral patterns, psychophysiological characteristics, gender-based disparities

## Abstract

Background/Objectives: The purpose of this study was to investigate whether or not there are notable differences between male and female vocational training students in terms of academic, behavioral, and psychophysiological characteristics. Methods: A total of 411 vocational training students, 210 of whom were male and 201 of whom were female, participated in an online survey that assessed academic, behavioral, and psychophysiological patterns. Results: In terms of extraversion and openness to experience, our research revealed that no statistically significant differences were noticed. When it came to agreeableness, however, significant differences were seen, with female students getting higher marks than male students. Furthermore, we found that there were significant gender differences in terms of neuroticism and conscientiousness, with female students achieving higher scores than male students. When compared to their male counterparts, females displayed higher performance in terms of academic success. They also demonstrated superior values in terms of reported stress, anxiety, and cognitive inflexibility. Conclusions: The findings of this study provide a more in-depth understanding of the numerous factors that have an impact on students engaging in vocational training. This will serve as a foundation for the development of individualized instructional and support programs.

## 1. Introduction

Vocational education and training (VET) is one of the learning programs that are gaining strength as an alternative to other higher studies after the end of basic and compulsory education throughout Europe and the world, such as in Latin America and the Asian region. In Spain (since its official incorporation in 1990), this type of training is gradually catching up with other leading countries in the European area in terms of qualifications and is one of the strong points of the European development agenda [1,2]. The demands of the productive sector and potential employers promote this training within the national administration as an attractive opportunity for students to acquire practical skills close to the world of work. Spain is one of the countries with the highest percentage of recent VET graduates benefiting from work-based learning during their VET program, at 98.4%, compared to the European average of 60.1% [3,4]. Similarly, the employment rate of young graduates is 64.6%, compared to the EU average of 57.6% [5,6]. 

These data show that in the past 5 years, there has been an increase in enrolment of almost 30%, with the number of students in vocational training in the education system in Spain exceeding one million (1,030,052 students), studying in 3921 training complexes in the Spanish State, according to data from the Ministry of Education. As far as the participation of women in these courses is concerned, there is a virtual parity with men in the middle and higher levels (41.5% and 48.9% respectively), and they represent around a third (29.5%) of students in basic vocational training. However, women continue to be in the majority in the occupational groups associated with personal image, socio-cultural and community services, textiles, clothing and leather goods, administration and management, and health [7].

At every stage of education, there are demands that can increase the anxiogenic state of the student [8]. However, the moment when students finish their compulsory education and begin to decide on their immediate future at a formative or working level is an important challenge where the demands of autonomy, self-management, and responsibility make the level of uncertainty, insecurity, and stress increase in a very noticeable way. In this respect, the lack of self-awareness, self-determination, and intrinsic or extrinsic motivation can determine the student’s commitment to the learning process [9,10]. Several of these problems have increased since the recent COVID-19 pandemic, with the young adult population experiencing perceptions of depression, feelings of loneliness [11], changes in communication habits, sedentary lifestyles, or an abuse of technological media, which can influence their self-perception of quality of life and academic performance [12]. In addition, vocational education provides students with many practical situations. These students are placed in challenging simulated situations that are close to actual work experience, which may cause students to feel pressure or insecurity when performing certain tasks or essential technical skills in their field of work. Other related factors could be relationships with peers or the classroom environment, learning difficulties, or adapting to change brought about by a standardized educational model [13,14].

In terms of gender differentiation, VET in Spain reflects enduring social norms and expectations. Males predominantly opt for technical sectors and prioritize a swift entry into the labor market, whereas females often select fields associated with caregiving and services. These gendered choices not only shape their educational and career trajectories but also impact their experiences and overall satisfaction within the VET system. Addressing these disparities requires sustained efforts through policies that promote gender diversity across all VET specializations, thereby fostering equity and challenging traditional stereotypes. Such initiatives are crucial for ensuring a more inclusive and balanced vocational education landscape, which can better serve the diverse needs of the workforce and society at large [15]. Previous research has shown some differences between women and men in terms of the level of perceived stress [16], anxiety before demanding situations at school, or coping ability, where women could have higher scores [17], but also in terms of affectivity and global emotional intelligence [18]. It has also been observed that there is a certain differentiation between the results in academic training and the transition to the working environment, where the need for a good atmosphere and a sense of belonging, and the need for control and dominance are established differently between women and men [19].

The objective of this study was to analyzed differences in academic, behavioral, and psychophysiological variables among male and female vocational training students. The initial hypothesis posits that there will be significant differences between male and female students in these variables, influenced by gender-specific traits and behaviors. This research aims to provide insights into the diverse factors that impact vocational training students, offering a basis for tailored educational and support strategies.

## 2. Materials and Methods

### 2.1. Participants

The study provided online questionnaires to a total of 411 participants, consisting of 210 males and 201 females. These participants were students enrolled in vocational education and training (VET) programs. Participants gave their complete names and email addresses throughout the registration process. This information was treated with the utmost confidentiality and managed in compliance with data privacy regulations. This study followed all guidelines for ethical human subjects research that are laid out in the 2013 Brazilian revision of the Helsinki Declaration. The research was carried out in compliance with existing ethical norms and the study was approved by the University Ethics Committee (CIPI/213006.55).

### 2.2. Procedures 

To fill out the survey, participants were given the option to either scan a QR code or use a specific Google Forms link. Before participating in this study, all subjects were fully informed of the aims and procedures of the study. The inclusion criteria for the study required participants to be enrolled in a vocational training program, to be aged between 16 and 30, and to provide informed consent (personal or legal tutor). They also needed to complete the online survey fully and provide demographic information, including gender, age, height, weight, and BMI. Exclusion criteria included incomplete or inconsistent survey responses, a lack of informed consent, non-enrollment in a vocational training program, being outside the specified age range, and any data breaches of confidentiality or privacy regulations. 

To ensure response reliability, each participant’s submission was checked for consistency and completeness. A preliminary analysis was conducted to identify any patterns of skewing or unusual responses that might indicate potential response bias. Participants were explicitly informed that their participation was completely voluntary and could withdraw at any point during the study without penalty or consequence. In order to participate, individuals were required to provide their digital consent by signing an informed consent form, thereby indicating their full understanding and willingness to participate in this research.

Age and anthropometric characteristics, including gender, height in centimeters, weight in kilograms, and body mass index in kilograms per square meter, were examined in the Google Forms questionnaire (BMI: kg/m^2^).

### 2.3. Measurements 

To analyze variables related to academic performance, the following parameters were evaluated: degree and course; average academic grade; and average grade in academic course 2022–2023.

#### 2.3.1. Behavioral Description

To analyze the participant’s behavioral factor, the following parameters were analyzed: hours of sleep per day; sleep quality perception (0–10); average daily steps; minutes of weekly aerobic activity training; minutes of weekly self-load activity training; hours spent watching TV per day; and hours spent using social media per day.

#### 2.3.2. Psychological Profile 

Various measures and inventories were used to assess the psychological traits in the study. The five personality traits—openness to experience, conscientiousness, extraversion, agreeableness, and neuroticism—were evaluated using the Spanish version of the Big Five Inventory, which had an alpha coefficient of 0.73. Using a 5-point Likert scale from 1 (totally disagree) to 5 (totally agree), the 44-item assessment was evaluated. We measured anxiety using a shortened version of the Spielberger State-Trait Anxiety Inventory (STAI) developed for the Spanish market, as presented in Ref. [20], consisting of six items that evaluated anxiety. Responses were rated on a 4-point Likert scale ranging from 1 (not at all) to 4 (very much), with an alpha coefficient of 0.93. In addition, a short Spanish version of the Perceived Stress Scale (PSS-4) [21] was used to assess participants’ perceived stress levels. The scale consisted of four items. Each item was answered on a five-point Likert scale ranging from 0 (never) to 4 (very often).

The study used the Spanish version of the Acceptance and Action Questionnaire II [22]. It assesses psychological rigidity or aversion to new experiences. There were seven questions on the survey, and respondents could choose from a seven-point Likert scale where zero means never and seven means always. This scale had an alpha coefficient of 0.84 (with a possible range of 0.78 to 0.88).

The Spanish version of the UCLA Loneliness Scale was used to measure loneliness, which had an alpha coefficient of 0.94 [23]. The condensed version of this scale consisted of three items, with responses rated on a 3-point Likert scale ranging from 1 (never) to 3 (frequently).

The ZUNG Depression Scale in Spanish was used [24]; during the COVID-19 pandemic, it was modified to evaluate depression. There are 20 items on this self-report measure, and they are both positive and negative. Its sensitivity and specificity are both above 80%, and its alpha coefficient is 0.09. The cognitive and somatic symptom dimensions of the measure each have eight items, whereas the mood and psychomotor dimensions each contain two.

### 2.4. Statistical Analysis

For the statistical analysis, the software SPSS version 24.0 was utilized. We began by determining the means and standard deviations of all the variables under study. The variables’ normality and homogeneity were checked using the Kolmogorov–Smirnov tests. The researchers used an independent *t*-test to look for differences between the research variables. A *p*-value of less than 0.05 was set as the threshold for statistical significance.

## 3. Results

Significant gender differences were found in height, weight, BMI, overall average score and average course grade (Table 1 and Table 2). 

Regarding the psychological profile, the variables indicate that there are statistically significant distinctions between the two groups (Table 3). The research indicated that women had a greater likelihood of experiencing elevated levels of anxiety (*p* = 0.00), cognitive inflexibility (*p* = 0.025), and emotional stress (*p* = 0.00). Nevertheless, there was no noticeable disparity in the level of depression displayed by each group. In the 2022–2023 school year, there were considerable discrepancies in academic achievement between males and females, with males consistently performing lower than females. The performance of girls was found to be generally higher than that of males. Regarding self-load activities, the sole distinction between the two groups in terms of physical activity is that males exhibit a greater level of enthusiasm when engaging in these activities (Table 4).

Related to psychological factors, outcomes indicated that women exhibit higher levels of agreeableness than men. Nevertheless, no statistically significant results were observed for extraversion or openness to experience. Similarly, notable gender disparities were observed in neuroticism and conscientiousness, with female students achieving higher scores. Stress, depression, and anxiety also presented higher outcomes in women; however, no significant differences were found in loneliness.

Regarding gender disparities in physical activity and lifestyle behaviors, it is evident that women have lower sleep quality, resulting in a more limited amount of rest compared to males. Simultaneously, women tend to engage in greater overall physical activity, particularly aerobic exercises such as walking, compared to males. However, they typically engage in fewer exercises that involve self-loading. Regarding the amount of time spent using social networks, women tend to spend more hours each day on them.

## 4. Discussion

The aim of this study is to examine gender disparities in academic achievement, behavior, and psychophysiological patterns among female students who are enrolled in vocational training programs. The main hypothesis was confirmed, as it was found that there are significant gender disparities in academic, behavioral, and psychophysiological patterns.

In terms of psychological factors, research indicates that women exhibit a higher degree of extroversion during interpersonal interactions than males [25]. However, this is not related to the results of our study, where no significant results were obtained for extraversion or openness to experience. Significant differences were found in agreeableness, where female students obtained higher values than male students. Similarly, significant gender differences were found in neuroticism and conscientiousness, where female students obtained higher values, in line with previous research [16,26,27]. Epidemiology, according to Leal et al. [28], refers to a higher frequency of anxiety and depression in female than in male participants, even mentioning that between 50 and 60% of cases are in females. Our findings are in line with this, because females have higher levels on both scales. This is possible because there is a strong comorbidity between the two types of disorders: many of the symptoms attributed to anxiety are also present in depression, and vice versa; however, there are differences that allow the two types to be identified [29,30,31,32]. These findings are consistent with those of prior investigations that examined the mental health of university students [33,34]. 

The development, maintenance, and exacerbation of a diverse array of psychological issues have been hypothesized to be influenced by psychological inflexibility and experiential avoidance [35,36]. For this reason, the results obtained in this study may be associated with anxiety, depression, and experiential avoidance, with higher levels in females than in males. Likewise, our findings are consistent with other research that emphasizes the significance of avoiding the rigid dominance of psychological reactions to the values and contingencies that are selected to govern action in life, or in other words, the importance of being psychologically flexible. Increased well-being and quality of life are positively correlated with greater psychological flexibility [37], whereas avoidance behaviors are directly proportional to psychological distress [38,39]. In this line, females reported significantly higher total levels of PSS than males. Being female has been positively associated with perceived stress [40,41], similar to our findings. Brougham et al. [42] also agreed in their research, where, in general, university women’s stress levels were higher. This susceptibility to stress levels in women was consistent with other studies [43,44,45]. Schmaus et al. [46] analyzed gender differences in a controlled setting and found that women were possibly more vulnerable to repeated exposures to stress compared to men [41]. Additionally, stress variables could be influenced by social media usage where females have shown higher values than males [47]. This fact was seen before, as previous studies pointed out that young women may be at a greater risk of experiencing everyday stress [48], engaging in problematic usage of social media, and having low levels of mental health [49]. In addition to this, variations might also arise from the level of physical exercise and activity engaged in [50,51]. Our study found that men engage in a higher frequency of self-loading workouts compared to women, whereas no disparities were observed in aerobic tasks. The efficacy of physical activity in mitigating stress and anxiety is well documented, suggesting that it may significantly contribute to the disparity in mental well-being between women and men [52]. This fact can also be related to the poor quality of sleep presented by our results; in this line, Nowakowski et al. showed that women tend to sleep worse [53].

Regarding academic achievements, researchers have long been intrigued by gender inequalities in academic attainment [54]. Historically, male students have outnumbered female students in science and technology fields and have generally rated their academic achievements higher [55,56]. In contrast, there is a common perception that nurses, for instance, are mostly female. The majority of nursing schools enroll mostly female students [57,58]. However, most studies found that the clinical placement satisfaction of male students was equivalent to that of females [57]. The idea that men are inherently smarter than women was perpetuated by the historical trend in men’s comparative advantage in academic performance [59]; however, cognitive psychology provides evidence that men and women have equal tested intellect, but there are prevalent gender disparities in self-estimated intelligence (SEI), where males consistently provide higher estimations than females [60]. Regarding vocational training, our findings indicate notable and significative differences in academic performance among vocational training students, with women consistently achieving superior scores overall, particularly in the final academic year of 2022–2023. In this line, the documented disparities in personality traits that are indicative of academic and professional achievement may provide insight into the underlying factors contributing to two contrasting gender disparities [61]. Currently, female students in both American and European countries exhibit superior academic performance compared to their male counterparts across all educational levels [62]. According to Spinath et al. (2014), girls have a greater graduation rate from higher school tracks compared to boys [63]. Additionally, female students now make up a larger percentage of those earning Bachelor’s and Master’s degrees [64,65]. Hence, when considering gender disparities in academic achievement, it is plausible to attribute them to individual student attributes such as intellect, temperament, and personality, as viewed through a psychological lens. In this regard, in nations committed to equal chances, females exhibit superior adaptability to the academic demands of school environments due to their intelligence, personality, and motivation [63]. In a recent study, Mammadov et al. shown that the combined influence of cognitive capacity and personality traits accounted for 27.8% of the variation in academic achievement. The predictor with the highest significance, accounting for 64% of the overall relevance, was cognitive ability. Conscientiousness was found to be a highly reliable and powerful indicator of performance [66], even after considering cognitive capacity. This factor is related to our results; this variable, as mentioned before, is higher in females. Generally, it explained 28% of the variability in academic achievement [67]. Multiple research studies have indicated that women exhibit a somewhat higher level of conscientiousness compared to men [66,68,69]. Currently, there is a lack of evidence-based interventions specifically designed to enhance conscientiousness. The existing research on boosting conscientiousness is primarily centered around clinical environments [69].

Despite the clear disparities identified, there remains a gap in evidence-based interventions tailored to enhance behavioral and psychological protocols, highlighting an area ripe for further research and intervention development. As we strive for gender equity in education, understanding these nuanced dynamics is paramount in crafting effective policies and interventions that empower all students to reach their full potential, irrespective of gender.

## 5. Limitations of the Study and Future Research Lines

Possible limitations of the study include sample bias, as the use of an online survey may have created selection bias. Self-report measures employed for evaluating variables may also be susceptible to response bias, which can potentially result in measurement inaccuracy. Furthermore, the questionnaires did not have information about the participants’ economic status, which may be taken into consideration. 

An essential aspect to address in future study, as emphasized by earlier authors, is the examination of environmental and behavioral factors, including those related to diet and dental health. In addition, biological measurements could offer the potential to quantify stress hormones such as cortisol, adrenaline, and alpha amylase.

## 6. Practical Applications

The practical applications stemming from the findings of this study are diverse and can be implemented in various educational and social settings to promote gender equity and enhance student well-being and academic success. Specific strategies to address gender disparities in mental health include the following.

1. Tailored Educational Interventions: Educational institutions can develop targeted interventions to address the specific mental health needs of male and female students. For instance, offering stress management workshops and mental health support services tailored to the higher levels of anxiety and depression observed among female students can help alleviate psychological distress. Similarly, providing workshops focused on anger management and emotional regulation for male students, who might experience higher levels of externalizing behaviors, can promote overall well-being.
2. Gender-Sensitive Teaching Strategies: Educators can incorporate gender-sensitive teaching strategies into their pedagogical approaches to create inclusive learning environments. This might involve adjusting teaching methods to recognize the different learning styles and preferences of male and female students. For example, using collaborative projects to engage female students and hands-on, problem-solving activities to capture the interest of male students can foster greater engagement and academic achievement.
3. Promotion of Psychological Flexibility: Given the association between psychological inflexibility and mental health issues observed in the study, promoting psychological flexibility can be beneficial. Integrating mindfulness practices and cognitive-behavioral techniques into educational curricula can help all students develop adaptive coping mechanisms. Special attention can be given to female students, who might benefit more from mindfulness practices to manage anxiety, while male students might benefit from cognitive-behavioral techniques that focus on changing thought patterns associated with stress and aggression.
4. Encouraging Physical Activity: Recognizing the role of physical activity in mitigating stress and anxiety, educational institutions can promote regular exercise and physical activity among students. Gender-sensitive approaches could include offering a variety of physical activities that appeal to different genders.
5. Personality Development Programs: Institutions can offer personality development programs aimed at enhancing conscientiousness and other positive personality traits associated with academic success. These programs can be tailored to address gender-specific challenges, such as fostering self-discipline and perseverance among male students who might struggle with impulsivity, and encouraging goal-setting and assertiveness among female students who might face societal pressures to conform to passive roles.
6. Policy Development: Policymakers can use the insights gained from this study to inform the development of gender-sensitive policies and initiatives within the education sector. This might include promoting gender equity in access to educational resources, addressing gender-based stereotypes and biases, and advocating for the implementation of supportive environments that foster the holistic development of all students. Ensuring that educational materials and curricula do not perpetuate gender stereotypes is crucial for creating an inclusive learning environment.
7. Hospital Interventions: Hospitals can implement gender-specific mental health support services for students. For example, establishing mental health clinics within hospitals that offer specialized counseling for female students dealing with anxiety and depression, and for male students addressing issues such as substance abuse or anger management. Additionally, training healthcare providers to recognize and address gender-specific mental health concerns can improve the quality of care and support provided to students.

Implementing these strategies can contribute to reducing gender disparities in mental health, promoting gender equity, and enhancing the overall well-being and academic success of students in vocational education and training programs in Spain.

## 7. Conclusions

This study highlights significant gender discrepancies in academic performance, behavioral characteristics, and psychophysiological patterns among female and male students enrolled in vocational training programs. While our data confirm the existence of these variations, they also reveal an intriguing complexity among psychological and academic components. Although agreeableness, neuroticism, and conscientiousness exhibited significant gender disparities, no notable gender differences were observed in terms of extraversion and openness to experience. The observation that female students exhibit higher degrees of anxiety and perceived stress aligns with epidemiological patterns, suggesting potential links between psychological inflexibility and the avoidance of specific situations. Moreover, these women exhibited elevated levels of perceived stress, maybe affected by results related to social media pressures and variations in individuals’ lack of physical exercise.

Additionally, this research specifically shows that females consistently outperform males academically, a pattern that is visible across various educational levels. These variations may arise not only from cognitive abilities but also from psychological characteristics, specifically conscientiousness, a feature frequently linked to females. This aligns with wider socioeconomic trends, where women are progressively outperforming males cognitively, surpassing them in terms of graduation rates and degrees earned. Nevertheless, despite the clear inconsistencies, there is still a lack of evidence-based interventions that expressly target the improvement of mental health in vocational training educational centers. This highlights an area ripe for additional exploration and the creation of solutions. In order to develop effective policies and interventions that enable all students to realize their full potential, it is crucial to have a comprehensive understanding of the intricate dynamics involved in achieving gender equality in education.

## Figures and Tables

**Table 1:** Demographic, education, and employment data.

					95% Confidence Interval of the Difference	
Variable	Male	Female	T	*p*	Lower	Upper
Age (Years)	21.1 ± 4.7	22.5 ± 6.9	−2.445	0.015	−2.583	−0.280
Height (cm)	177.9 ± 7.5	163.1 ± 6.8	20.898	0.000	13.445	16.237
Weight (Kg)	73.2 ± 12.2	58.1 ± 10.7	13.271	0.000	12.879	17.358
Body Mass Index (kg/m²)	23.1 ± 3.4	21.5 ± 4.0	4.282	0.000	0.853	2.301
Course	1.2 ± 0.4	1.2 ± 0.4	−1.328	0.185	−0.127	0.024
Satisfaction with studies	3.9 ± 0.8	4.0 ± 0.8	−1.421	0.156	−0.274	0.044

**Table 2:** Academic performance.

					95% Confidence Interval of the Difference	
Variable	Male	Female	T	*p*	Lower	Upper
Overall average score	7.1 ± 1.4	7.5 ± 1.2	−2.930	0.004	−0.619	−0.122
Average course grade	7.2 ± 1.3	7.5 ± 1.2	−2.808	0.005	−0.596	−0.105

**Table 3:** Psychology and body satisfaction data.

					95% Confidence Interval of the Difference	
Variable	Male	Female	T	*p*	Lower	Upper
Extraversion (Big Five)	5.5 ± 1.8	5.4 ± 1.8	0.754	0.451	−0.211	0.475
Agreeableness (Big Five)	5.8 ± 1.5	6.2 ± 1.6	−2.927	0.004	−0.754	−0.148
Conscientiousness (Big Five)	6.2 ± 1.7	6.6 ± 1.8	−2.251	0.025	−0.714	−0.048
Neuroticism (Big Five)	5.2 ± 1.9	6.9 ± 1.9	−8.671	0.000	−2.051	−1.293
Openness to Experience (Big Five)	7.4 ± 1.8	7.3 ± 1.8	0.528	0.597	−0.251	0.437
STAI	12.4 ± 3.7	13.8 ± 3.9	−3.761	0.000	−2.162	−0.677
AAQ II	22.3 ± 9.7	26.1 ± 10.3	−3.867	0.000	−5.730	−1.867
UCLA	4.4 ± 1.5	4.6 ± 1.7	−1.739	0.083	−0.584	0.035
PSS4	5.6 ± 3.2	6.8 ± 3.3	−3.656	0.000	−1.834	−0.551
ZUNG Score	47.7 ± 5.5	49.9 ± 5.7	−4.102	0.000	−3.342	−1.176

AAQ II (Acceptance and Action Questionnaire II); UCLA (UCLA Loneliness Scale); STAI (Spielberger State-Trait Anxiety Inventory); ZUNG (ZUNG Depression Scale); PSS4 (Perceived Stress Scale).

**Table 4:** Health and physical activity data.

					95% Confidence Interval of the Difference	
Variable	Male	Female	T	*p*	Lower	Upper
Daily hours of sleep	6.8 ± 1.1	6.8 ± 1.1	−0.454	0.650	−0.261	0.163
Sleep quality	6.1 ± 2.2	5.8 ± 2.0	1.497	0.135	−0.096	0.713
Daily hours of TV viewing	0.9 ± 0.9	0.9 ± 1.1	−0.635	0.526	−0.269	0.137
Daily hours of social network use	2.3 ± 1.6	2.8 ± 1.9	−3.212	0.001	−0.904	−0.217
Average daily steps	8550.4 ± 7269.3	9392.3 ± 23,792.7	−0.452	0.651	−4502.929	2819.095
Minutes of weekly aerobic training	150.9 ± 203.9	168.9 ± 197.0	−0.718	0.473	−67.203	31.270
Minutes of weekly self-load training	226.2 ± 296.8	129.4 ± 150.6	2.961	0.003	32.389	161.103

Sleep quality: scale of 0 to 10 (0 very poor sleep quality and 10 maximum sleep quality).

## Data Availability

Data are contained within the article.
